# *Lactobacillus helveticus* HY7804 Alleviates Ethanol-Induced Liver Injury and Modulates Oxidative Stress and Inflammatory Responses in a Chronic-Binge Ethanol-Fed Mouse Model

**DOI:** 10.3390/ijms27188058

**Published:** 2026-09-10

**Authors:** Hyeonji Kim, Daehyeop Lee, Haeryn Jeong, Joo-Yun Kim, Jae-Jung Shim, Jae-Hwan Lee

**Affiliations:** R&BD Center, hy Co., Ltd., 22 Giheungdanji-ro 24 Beon-gil, Giheung-gu, Yongin-si 17086, Republic of Korea; skyatk94@gmail.com (H.K.); flywhy7@hy.co.kr (D.L.); haeryn@hy.co.kr (H.J.); jjshim@hy.co.kr (J.-J.S.); jaehwan@hy.co.kr (J.-H.L.)

**Keywords:** alcohol-associated liver disease (ALD), probiotics, *Lactobacillus helveticus*, inflammation, oxidative stress, antioxidant

## Abstract

Alcohol-associated liver disease (ALD) contributes substantially to the global burden of chronic liver disease, yet few effective pharmacological treatments are currently available. The hepatoprotective potential of *Lactobacillus helveticus* HY7804 was evaluated in ethanol-exposed HepG2 cells and in a modified chronic-binge ethanol-fed mouse model adapted from the National Institute on Alcohol Abuse and Alcoholism (NIAAA) paradigm. Ethanol-induced cytotoxicity in HepG2 cells was attenuated by HY7804, as demonstrated by the recovery of cell viability and the reduction in lactate dehydrogenase release. HY7804 also decreased ethanol-induced inflammatory cytokine production, including IL-6, TNF-α, IL-1β, and IL-8, and modulated apoptosis-related gene expression. In chronic-binge ethanol-fed mice, HY7804 reduced serum AST from 183.4 ± 34.1 to 129.4 ± 16.4 U/L (*p* < 0.01) and ALT from 67.5 ± 4.5 to 52.1 ± 4.7 U/L (*p* < 0.05). Hepatic lipid accumulation was also significantly reduced by HY7804 treatment compared with the EtOH group, as indicated by the decreased Oil Red O-positive area (*p* < 0.01). Hepatic MDA levels decreased from 46.01 ± 3.38 to 30.58 ± 6.38 nmol/mg tissue (*p* < 0.01). In addition, HY7804 modulated hepatic inflammatory cytokine profiles, antioxidant-related responses, and the *Bax*/*Bcl-2* expression ratio. These findings suggest that HY7804 mitigates ethanol-induced hepatic injury, with associated changes in inflammatory responses, antioxidant-related markers, lipid peroxidation, and apoptosis-related gene expression.

## 1. Introduction

Alcohol-associated liver disease (ALD) is a major cause of chronic liver disease worldwide and encompasses a broad spectrum of hepatic disorders ranging from simple steatosis to alcohol-associated steatohepatitis, fibrosis, cirrhosis, and liver failure. The global prevalence of ALD has been estimated at approximately 3.5% in the general population, 26.0% among hazardous drinkers, and 55.1% among individuals with alcohol use disorder, highlighting its substantial epidemiological burden [1,2]. Although alcohol abstinence remains the most effective strategy for preventing ALD progression, effective pharmacological options remain limited, and current management largely relies on abstinence and supportive care [3]. Therefore, novel preventive or adjunctive approaches targeting the multifactorial pathogenesis of alcohol-associated hepatic damage are needed.

Ethanol-induced hepatic injury involves dysregulation of redox balance and inflammatory responses associated with alcohol metabolism [4,5]. Ethanol metabolism via cytochrome P450 2E1 (CYP2E1) contributes substantially to reactive oxygen species (ROS) production, which can promote hepatic oxidative stress and compromise antioxidant defenses [6,7]. This oxidative imbalance contributes to lipid peroxidation and oxidative damage to cellular macromolecules, while also impairing antioxidant defense systems. In particular, sustained oxidative stress may affect Nrf2/HO-1-mediated antioxidant responses, potentially influencing hepatic defense mechanisms against ethanol-induced oxidative injury [8]. Along with oxidative disturbances, the inflammatory response associated with alcohol exposure involves NF-κB-related signaling and the subsequent production of pro-inflammatory cytokines in the liver [4,9]. Thus, interventions that restore redox balance and suppress excessive inflammatory responses may represent promising strategies for preventing alcohol-induced hepatic damage.

Experimental animal models are essential for investigating alcohol-associated hepatic alterations and evaluating candidate hepatoprotective agents. Among these models, the National Institute on Alcohol Abuse and Alcoholism (NIAAA) chronic-binge ethanol feeding model is commonly used as an experimental approach for evaluating ethanol-induced hepatic injury and related pathological changes [10,11]. This model combines chronic ethanol feeding using a Lieber-DeCarli ethanol liquid diet with a single ethanol binge, thereby inducing hepatic steatosis, inflammation, and serum transaminase elevation within a relatively short experimental period. In addition, this model partially recapitulates acute-on-chronic alcohol-induced liver injury by combining repeated ethanol exposure with a binge ethanol challenge, thereby reflecting clinically relevant drinking patterns observed in patients with ALD [10,12,13]. Female mice have been reported to be more susceptible to alcohol-induced liver injury than male mice, providing a rationale for their use in the chronic-binge ethanol feeding model [10,14].

Probiotics are defined as live microorganisms that confer a health benefit on the host when consumed in adequate amounts [15]. Among probiotic species, *Lactobacillus helveticus* is a Gram-positive, thermophilic, homofermentative lactic acid bacterium traditionally used as a starter or adjunct culture in dairy fermentation [16,17]. *L. helveticus* strains have been reported to exhibit various health-promoting properties, including host immune modulation, inhibition of pathogens, and gut microbiota-related effects [18]. Previous studies suggest that probiotics may exert protective effects against ethanol-associated hepatic injury, potentially involving changes in oxidative stress, inflammatory responses, NF-κB-related signaling, and the gut microbiota [13,19,20,21].

*Lactobacillus helveticus* HY7804 (HY7804) was originally isolated from raw milk and has previously been investigated for its hepatoprotective potential in experimental models of metabolic dysfunction-associated steatotic liver disease (MASLD), formerly referred to as non-alcoholic fatty liver disease (NAFLD). In a diet-induced MASLD mouse model, HY7804 improved liver injury-related markers, attenuated hepatic steatosis, and reduced histopathological features associated with hepatic damage [22]. More recently, HY7804 was reported to alleviate MASLD-associated hepatic alterations, accompanied by changes in intestinal barrier function, intestinal inflammation, and gut microbiota composition [23]. These previous findings suggest that HY7804 possesses beneficial properties related to hepatic lipid metabolism, inflammation regulation, and host–microbiota interactions, which are also considered important factors in alcohol-associated hepatic injury.

Although HY7804 has shown hepatoprotective potential in MASLD models, its protective efficacy in ethanol-induced hepatic injury has not been investigated. Given that oxidative stress, inflammatory activation, and lipid dysregulation are common pathological features shared between metabolic and alcohol-related liver injury, previous evidence linking HY7804 to hepatic lipid metabolism and inflammatory responses supports further investigation of its effects under ethanol exposure conditions.

In the present study, we hypothesized that HY7804 could alleviate ethanol-induced hepatic injury by modulating key pathological responses associated with alcohol exposure. To test this hypothesis, we investigated the protective effects of HY7804 using an ethanol-exposure model in HepG2 cells and a modified mouse model incorporating chronic-binge ethanol feeding based on the NIAAA paradigm. This study aimed to determine whether HY7804 improves ethanol-associated hepatic alterations and to characterize its effects on inflammatory responses, antioxidant-related responses, lipid peroxidation, and apoptosis-associated changes.

## 2. Results

### 2.1. Effects of HY7804 on Cell Cytotoxicity

**Figure 1:**
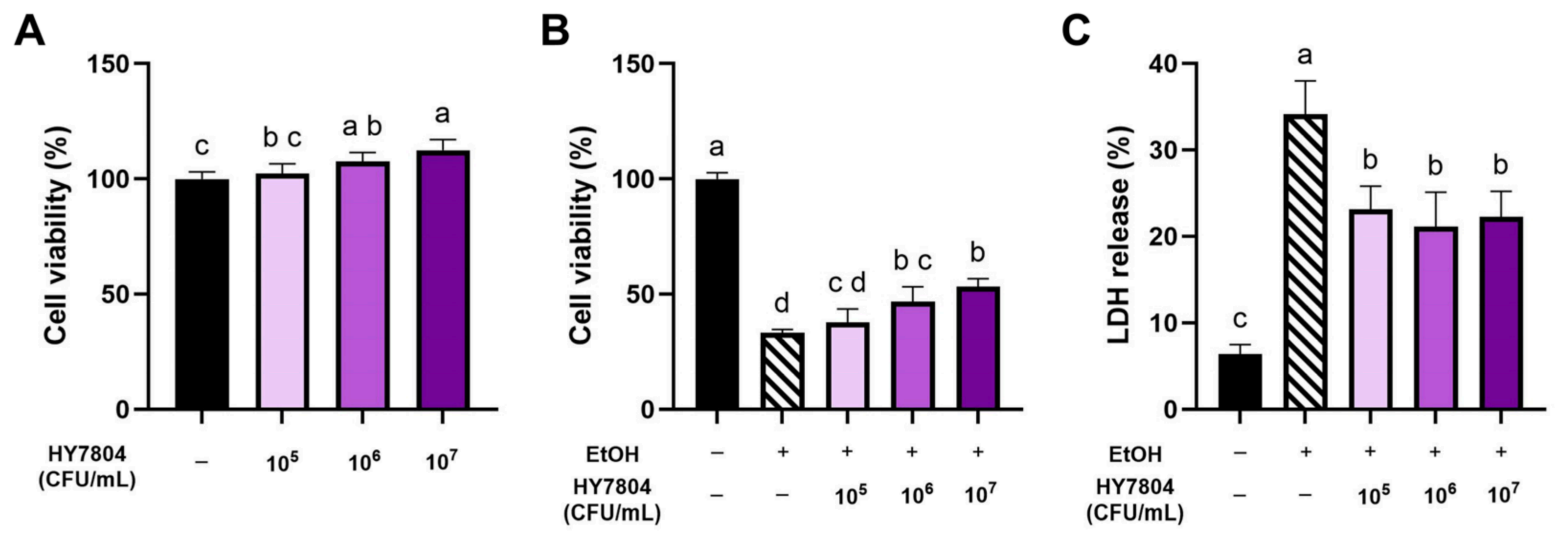
Effects of HY7804 on ethanol-associated cytotoxicity in HepG2 cells. (**A**) Cell viability following HY7804 treatment alone, (**B**) Cell viability, and (**C**) LDH release following ethanol exposure with or without HY7804 treatment. Data are shown as mean ± SD. Statistical comparisons were performed by one-way ANOVA with Tukey’s multiple comparisons test. Groups sharing at least one letter do not differ significantly, whereas groups with no letters in common differ significantly (*p* < 0.05). EtOH, ethanol; HY7804, *L. helveticus* HY7804; LDH, lactate dehydrogenase.

Cell viability and lactate dehydrogenase (LDH) release were measured to evaluate the cytotoxicity of HY7804 and its protective effects against ethanol-induced injury in HepG2 cells (Figure 1). The cell viabilities of HepG2 cells treated with 10^5^, 10^6^, and 10^7^ CFU/mL of HY7804 were 102.33%, 107.54% and 112.41%, respectively, indicating that HY7804 did not reduce HepG2 cell viability at the tested concentrations. Ethanol treatment significantly reduced cell viability to 33.27% (*p* < 0.001), whereas pretreatment with HY7804 at concentrations of 10^5^, 10^6^, and 10^7^ CFU/mL increased cell viability to 37.75% (not significant), 46.70% (*p* < 0.01) and 53.35% (*p* < 0.001), respectively. Figure 1C shows that ethanol exposure markedly elevated LDH release in HepG2 cells (*p* < 0.001), indicating increased cytotoxicity. Pretreatment with HY7804 significantly reduced LDH release compared with ethanol-treated cells (*p* < 0.001). A bacteria-only control performed in the absence of HepG2 cells showed negligible MTT-derived absorbance under the same assay conditions, indicating minimal contribution of HY7804 itself to formazan formation (Table S1).

### 2.2. Effects of HY7804 on Secretion of Pro-Inflammatory Cytokines in HepG2 Cells

**Figure 2:**
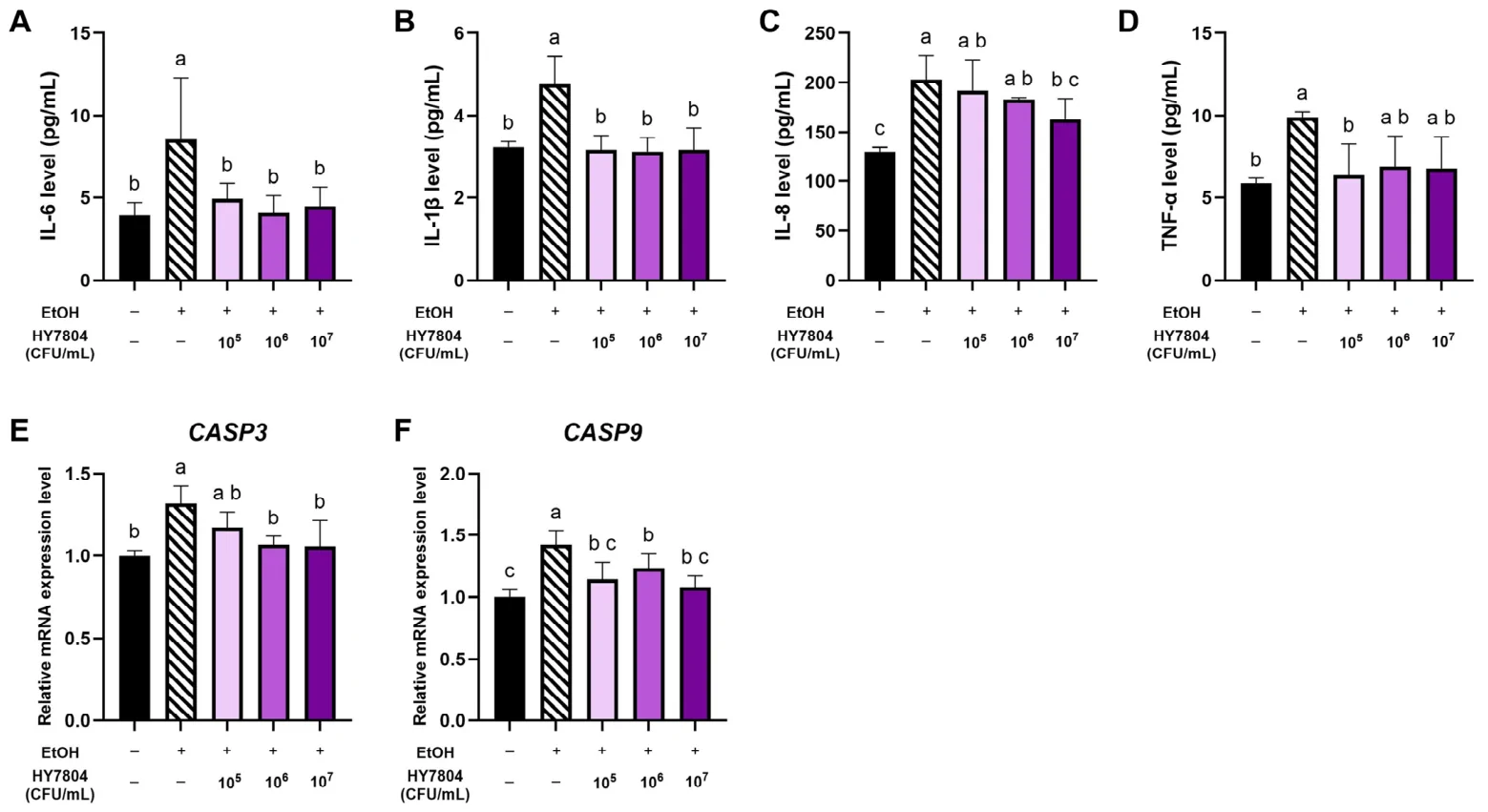
Cytokine responses and apoptosis-related gene expression following HY7804 treatment in ethanol-exposed HepG2 cells. (**A**–**D**) Pro-inflammatory cytokine secretion was evaluated by measuring IL-6 (**A**), IL-1β (**B**), IL-8 (**C**), and TNF-α (**D**). (**E**,**F**) The expression levels of the apoptosis-related genes *CASP3* (**E**) and *CASP9* (**F**) were determined. Data are presented as mean ± SD. Statistical comparisons were performed using one-way ANOVA followed by Tukey’s multiple comparisons test. Groups sharing at least one letter do not differ significantly, whereas groups with no letters in common differ significantly (*p* < 0.05). EtOH, ethanol; HY7804, *L. helveticus* HY7804; IL-6, interleukin-6; IL-1β, interleukin-1 beta; IL-8, interleukin-8; TNF-α, tumor necrosis factor-alpha; *CASP3*, caspase-3; *CASP9*, caspase-9.

The effects of HY7804 on ethanol-associated cytokine responses were evaluated by measuring IL-6, IL-1β, IL-8, and TNF-α secretion in HepG2 cells (Figure 2A–D). The secretion levels of IL-6, IL-1β, IL-8, and TNF-α were significantly elevated in EtOH-treated HepG2 cells compared with untreated cells (IL-6, IL-1β, and IL-8, *p* < 0.001; TNF-α, *p* < 0.05). Pretreatment with HY7804 (10^5^–10^7^ CFU/mL) significantly suppressed the levels of IL-6 and IL-1β. IL-8 levels decreased in a dose-dependent manner following HY7804 treatment, with a significant reduction observed only at 10^7^ CFU/mL (*p* < 0.05). TNF-α levels were also reduced following HY7804 pretreatment, with similar reductions observed across all tested concentrations.

### 2.3. Effect of HY7804 on Apoptosis-Related Gene Expression in HepG2 Cells

The mRNA expression levels of apoptosis-related genes were evaluated in HepG2 cells following ethanol exposure (Figure 2E,F). Compared with untreated cells, ethanol treatment significantly increased the expression of *CASP3* and *CASP9* by 1.31-fold and 1.42-fold, respectively (*p* < 0.001). HY7804 treatment resulted in a reduction in *CASP3* expression at all tested concentrations. No significant change was observed at 10^5^ CFU/mL (1.20-fold), whereas significant decreases were detected at 10^6^ and 10^7^ CFU/mL, with expression levels of 1.13- and 1.11-fold, respectively (*p* < 0.01). *CASP9* expression in HY7804-treated cells significantly decreased to 1.14- (*p* < 0.01), 1.23- (*p* < 0.05) and 1.08-fold (*p* < 0.001) at 10^5^, 10^6^ and 10^7^ CFU/mL, respectively.

### 2.4. Effects of HY7804 on Physiological Parameters, Organ Weights and Serum Liver Injury Markers in Chronic-Binge Ethanol-Fed Mice

**Figure 3:**
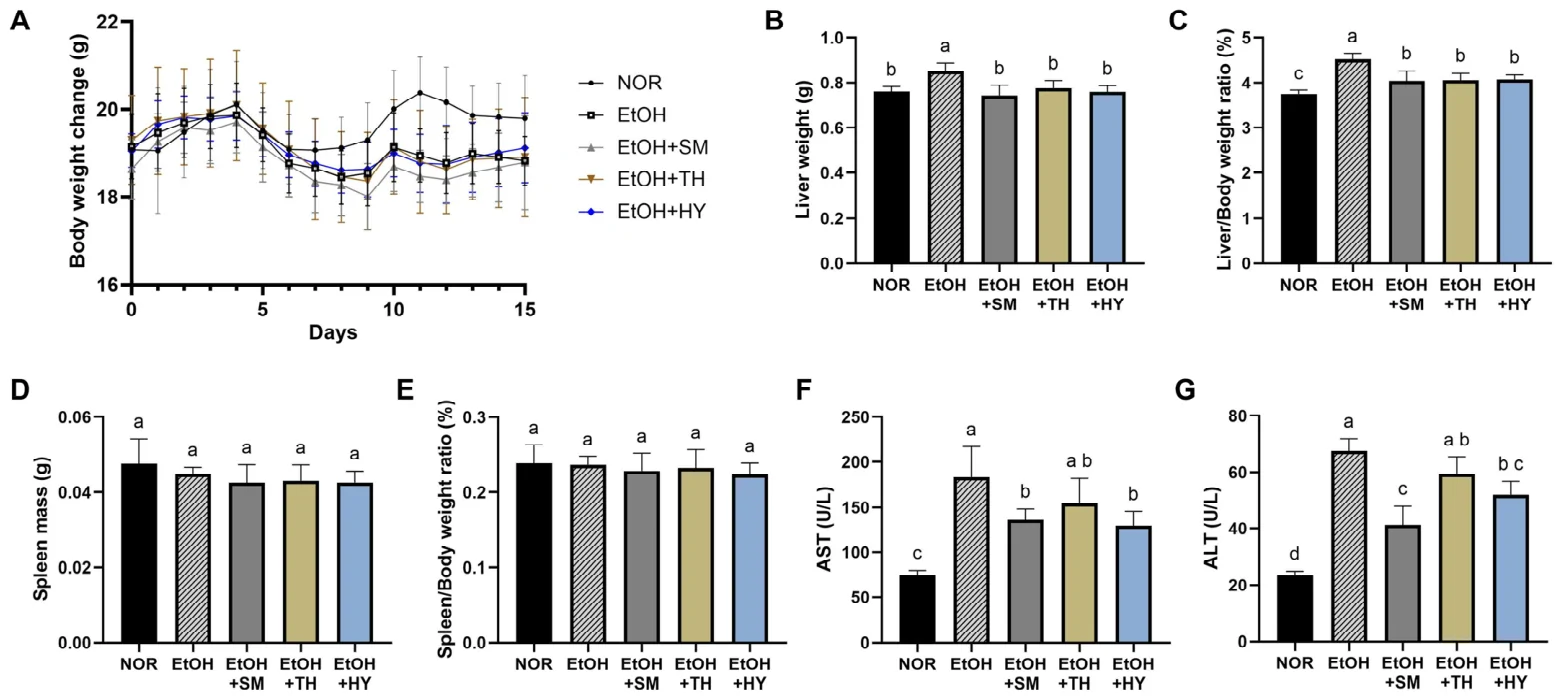
Physiological and serum biochemical responses to HY7804 treatment in chronic-binge ethanol-fed mice. Panels show changes in body weight over the experimental period (**A**), absolute and relative liver weights (**B**,**C**), absolute and relative spleen weights (**D**,**E**), and serum AST and ALT levels (**F**,**G**), respectively. Data are shown as mean ± SD. Statistical comparisons were performed using one-way ANOVA followed by Tukey’s multiple comparisons test. Groups sharing at least one letter do not differ significantly, whereas groups with no letters in common differ significantly (*p* < 0.05). AST, aspartate aminotransferase; ALT, alanine aminotransferase; NOR, normal group; EtOH, ethanol-fed group; EtOH + SM, silymarin-treated ethanol-fed group; EtOH + TH, *L. helveticus* type strain ATCC15009-treated ethanol-fed group; EtOH + HY, *L. helveticus* HY7804-treated ethanol-fed group.

The effects of HY7804 administration on body weight and liver and spleen weights were evaluated in mice subjected to chronic-binge ethanol feeding. Given its established hepatoprotective effects and reported antioxidant and anti-inflammatory activities, a silymarin (SM) treatment group was included as the positive control. Its reported low toxicity and long-standing use in liver disorders support its application as a reference agent in experimental liver injury models [13,24]. Ethanol feeding reduced body weight compared with the NOR group, whereas SM, TH, and HY7804 treatment did not significantly affect body weight compared with the EtOH group (Figure 3A). Ethanol feeding significantly increased liver weight and the liver-to-body weight ratio compared with the NOR group (*p* < 0.001). Treatment with SM, TH, or HY7804 resulted in significantly lower values for both parameters relative to the EtOH group (*p* < 0.001). In contrast, no significant group-dependent changes were observed in spleen weight or the spleen-to-body weight ratio (Figure 3D,E).

Analysis of serum biochemical parameters revealed that chronic-binge ethanol administration altered hepatic injury markers. Specifically, the EtOH group showed higher serum AST and ALT concentrations than the NOR group (Figure 3F,G; *p* < 0.001). Treatment with SM and HY7804 partially reversed these ethanol-associated changes, with significantly lower AST levels observed in intervention groups compared with the EtOH group (*p* < 0.05 and *p* < 0.01, respectively). Similarly, ALT levels were significantly decreased by SM and HY administration compared with the EtOH group (*p* < 0.001 and *p* < 0.05, respectively). Although AST and ALT levels were numerically lower in the EtOH + TH group than in the EtOH group. However, no statistically significant differences were detected. The reduction in serum transaminase levels following HY7804 administration was consistent with an attenuation of ethanol-associated hepatic injury in chronic-binge ethanol-fed mice.

### 2.5. Effects of HY7804 on Hepatic Histopathological Changes and Lipid Accumulation in Chronic-Binge Ethanol-Fed Mice

**Figure 4:**
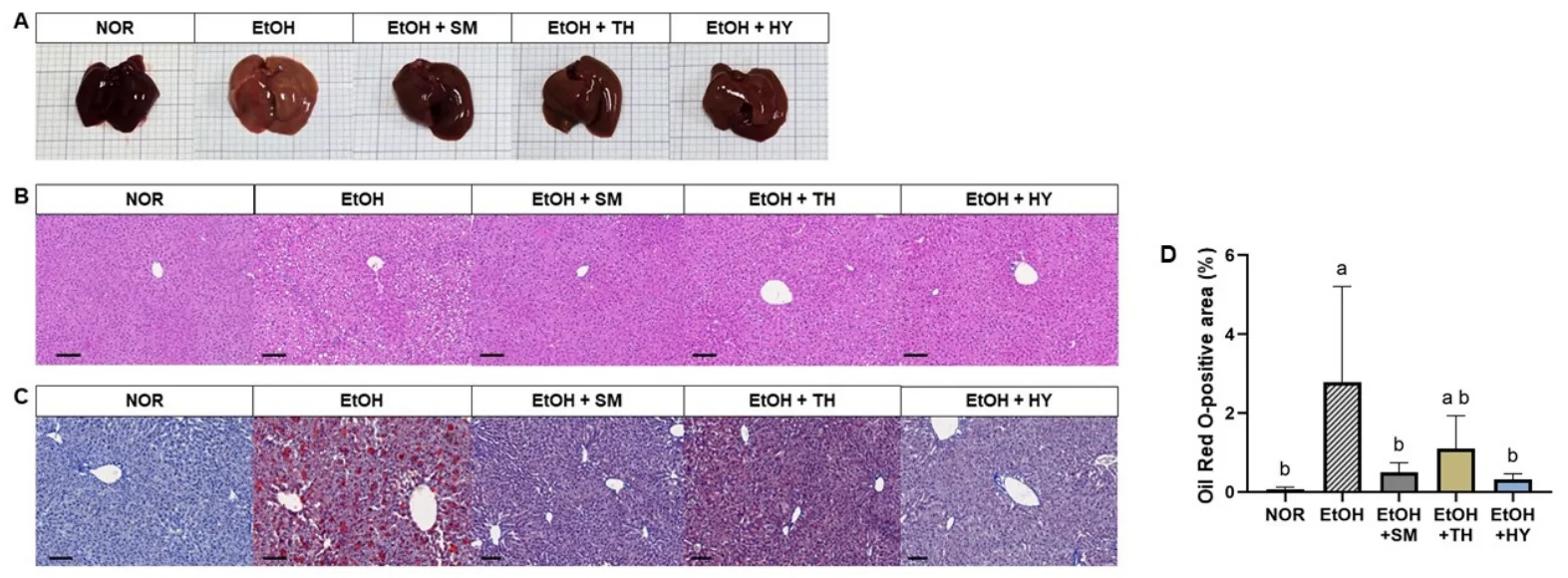
Effects of HY7804 on hepatic histopathological changes and lipid accumulation in chronic-binge ethanol-fed mice. (**A**) Liver tissue morphology. (**B**) Hematoxylin and eosin (H&E)-stained section images (100× magnification), (**C**) Oil Red O-stained section images (100× magnification). (**D**) Quantification of Oil Red O-positive area. Scale bar = 100 μm. Data are shown as mean ± SD. Statistical comparisons were performed using one-way ANOVA followed by Tukey’s multiple comparisons test. Groups sharing at least one letter do not differ significantly, whereas groups with no letters in common differ significantly (*p* < 0.05). NOR, normal group; EtOH, ethanol-fed group; EtOH + SM, silymarin-treated ethanol-fed group; EtOH + TH, *L. helveticus* type strain ATCC15009-treated ethanol-fed group; EtOH + HY, *L. helveticus* HY7804-treated ethanol-fed group.

Histological analyses were performed to determine whether HY7804 attenuates ethanol-induced hepatic structural damage and lipid accumulation. Macroscopic observation of excised livers showed that the liver size appeared increased in the EtOH group compared with the NOR group, whereas SM, TH, and HY7804 administration visibly reduced this ethanol-induced liver enlargement (Figure 4A). These gross morphological findings were accompanied by alterations in liver weight and liver-to-body weight ratio.

H&E-stained liver sections from the NOR group showed normal hepatic architecture with well-preserved hepatocyte morphology (Figure 4B). In contrast, the EtOH group exhibited histopathological alterations, including hepatocellular vacuolation and steatotic changes. These alterations were attenuated in the SM, TH, and HY7804-treated groups, with the HY7804 group showing improved hepatic morphology compared with the EtOH group.

Oil Red O staining further confirmed ethanol-induced hepatic lipid accumulation. The EtOH group showed a marked increase in Oil Red O-positive lipid droplets compared with the NOR group, whereas HY7804 administration reduced hepatic lipid deposition compared with the EtOH group. A similar decreasing trend was observed in the SM group. In Figure 4D, quantitative analysis of Oil Red O-positive areas showed that ethanol feeding significantly increased hepatic lipid accumulation relative to the NOR group (*p* < 0.01), while HY7804 significantly reduced this increase compared with the EtOH group (*p* < 0.01). Overall, these findings indicate that HY7804 alleviated chronic-binge ethanol-induced hepatic enlargement, lipid accumulation, and histopathological injury.

### 2.6. Effects of HY7804 on Hepatic Antioxidant Defense and Lipid Peroxidation in Chronic-Binge Ethanol-Fed Mice

**Figure 5:**
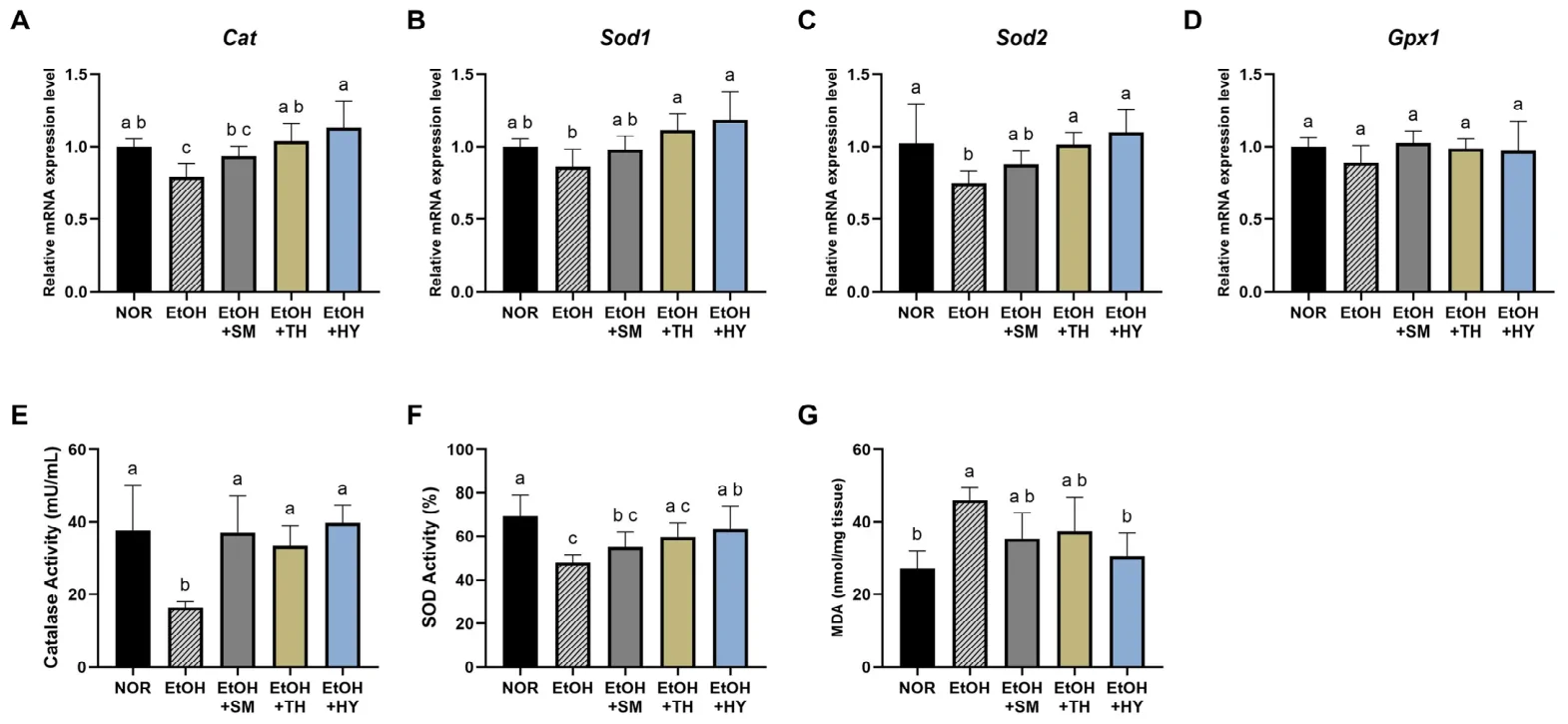
Effects of HY7804 on hepatic antioxidant responses in chronic-binge ethanol-fed mice. Relative mRNA expression of antioxidant-related genes (**A**) *Cat*, (**B**) *Sod1*, (**C**) *Sod2*, and (**D**) *Gpx1*. (**E**) hepatic catalase activity, (**F**) hepatic SOD activity, and (**G**) hepatic MDA concentration. Data are shown as mean ± SD. Statistical comparisons were performed using one-way ANOVA followed by Tukey’s multiple comparisons test. Groups sharing at least one letter do not differ significantly, whereas groups with no letters in common differ significantly (*p* < 0.05). *Cat*, catalase; *Sod1*, superoxide dismutase 1; *Sod2*, superoxide dismutase 2; *Gpx1*, glutathione peroxidase 1; MDA, malondialdehyde; NOR, normal group; EtOH, ethanol-fed group; EtOH + SM, silymarin-treated ethanol-fed group; EtOH + TH, *L. helveticus* type strain ATCC15009-treated ethanol-fed group; EtOH + HY, *L. helveticus* HY7804-treated ethanol-fed group.

The effects of HY7804 on ethanol-associated oxidative imbalance were assessed by analyzing hepatic antioxidant enzyme expression and activity together with lipid peroxidation in chronic-binge ethanol-fed mice. As presented in Figure 5A–C, ethanol feeding resulted in lower hepatic mRNA levels of *Cat*, *Sod1*, and *Sod2*, corresponding to 0.79-, 0.86-, and 0.72-fold of the NOR group, respectively. The reductions in *Cat* and *Sod2* were statistically significant (*p* < 0.05). HY7804 administration attenuated these ethanol-associated changes, increasing the expression of *Cat*, *Sod1*, and *Sod2* relative to the EtOH group (*Cat*, *p* < 0.001; *Sod1* and *Sod2*, *p* < 0.01). The respective expression levels reached 1.13-, 1.19-, and 1.09-fold of those measured in the NOR group. TH treatment produced a similar response, with *Cat*, *Sod1*, and *Sod2* expression reaching 1.04-, 1.11-, and 1.00-fold of the NOR values, respectively (*Cat*, *p* < 0.01; *Sod1* and *Sod2*, *p* < 0.05). SM administration also tended to increase the expression of antioxidant enzyme-related genes relative to the EtOH group, although these differences were not statistically significant. In contrast, *Gpx1* mRNA levels remained comparable across all experimental groups (Figure 5D).

As shown in Figure 5E,F, hepatic catalase and SOD activities were lower in ethanol-fed mice than in the NOR group (*p* < 0.001). SM, TH, and HY7804 administration was associated with increased catalase activity relative to the EtOH group, while a significant increase in SOD activity was observed only following HY7804 treatment (*p* < 0.05). The EtOH group exhibited a marked increase in hepatic malondialdehyde (MDA) levels compared with the NOR group (46.01 ± 3.38 vs. 27.02 ± 5.05 nmol/mg tissue, respectively; *p* < 0.001), consistent with increased lipid peroxidation. In contrast, HY7804 administration significantly reduced hepatic MDA levels to 30.58 ± 6.38 nmol/mg tissue compared with the EtOH group (Figure 5G; *p* < 0.05).

### 2.7. Effect of HY7804 on Hepatic Nrf2-Related Antioxidant Gene Expression in Chronic-Binge Ethanol-Fed Mice

**Figure 6:**
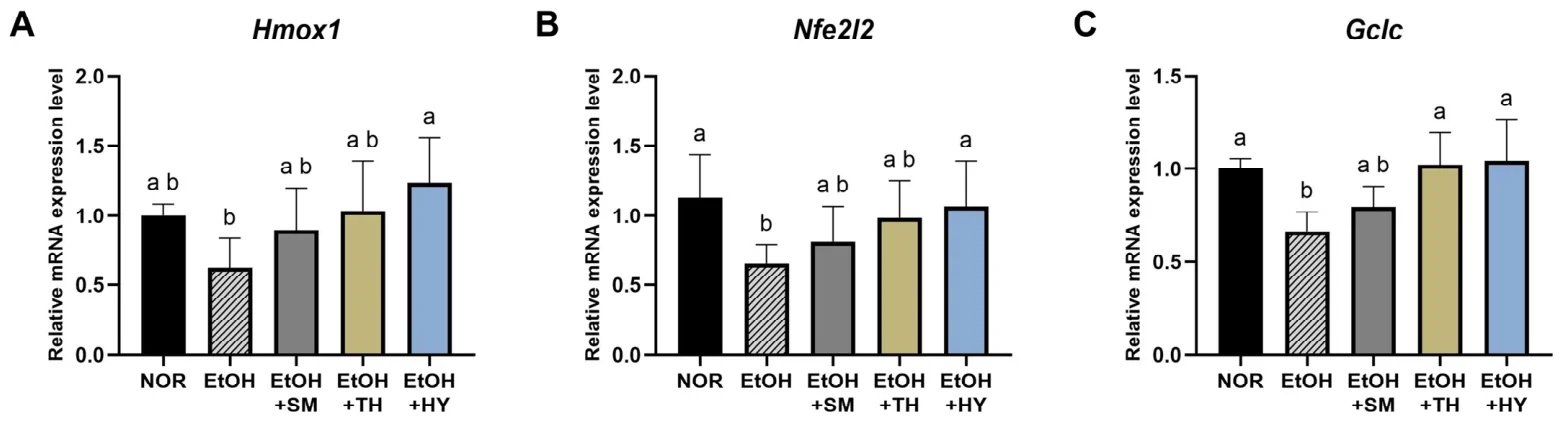
Hepatic expression of Nrf2-associated antioxidant genes in chronic-binge ethanol-fed mice following HY7804 administration. Relative mRNA levels of *Hmox1* (**A**), *Nfe2l2* (**B**), and *Gclc* (**C**) are shown. Data are shown as mean ± SD. Statistical comparisons were performed using one-way ANOVA followed by Tukey’s multiple comparisons test. Groups sharing at least one letter do not differ significantly, whereas groups with no letters in common differ significantly (*p* < 0.05). *Hmox1*, Heme oxygenase 1; *Nfe2l2*, Nuclear factor erythroid 2-related factor 2; *Gclc*, Glutamate-cysteine ligase catalytic subunit; NOR, normal group; EtOH, ethanol-fed group; EtOH + SM, silymarin-treated ethanol-fed group; EtOH + TH, *L. helveticus* type strain ATCC15009-treated ethanol-fed group; EtOH + HY, *L. helveticus* HY7804-treated ethanol-fed group.

To further assess the effect of HY7804 on hepatic antioxidant responses, the mRNA expression of *Hmox1*, *Nfe2l2*, and *Gclc* was analyzed in chronic-binge ethanol-fed mice. Hepatic *Hmox1* expression tended to decrease in the EtOH group to 0.62-fold of the NOR group, with no significant difference detected between the two groups. *Hmox1* expression was significantly increased in the EtOH + HY group to 1.23-fold of the NOR group compared with the EtOH group (Figure 6A; *p* < 0.01). Ethanol feeding significantly downregulated hepatic *Nfe2l2* and *Gclc* expression to 0.59- and 0.66-fold of the NOR group, respectively (*p* < 0.01). HY7804 administration significantly restored the expression of both genes compared with the EtOH group, with *Nfe2l2* and *Gclc* expression reaching 1.06- and 1.04-fold of the NOR group, respectively (Figure 6B,C; *p* < 0.05). TH also significantly restored *Gclc* expression to 1.02-fold of the NOR group (*p* < 0.05), whereas SM showed only non-significant increasing trends in these antioxidant response-related genes. Thus, these findings suggest that HY7804 improved ethanol-impaired hepatic antioxidant response-related gene expression, with a numerically favorable recovery pattern among the treatment groups, although no significant differences were observed among the SM, TH, and HY groups.

### 2.8. Effects of HY7804 on Hepatic Inflammatory Cytokine Levels in Chronic-Binge Ethanol-Fed Mice

**Figure 7:**
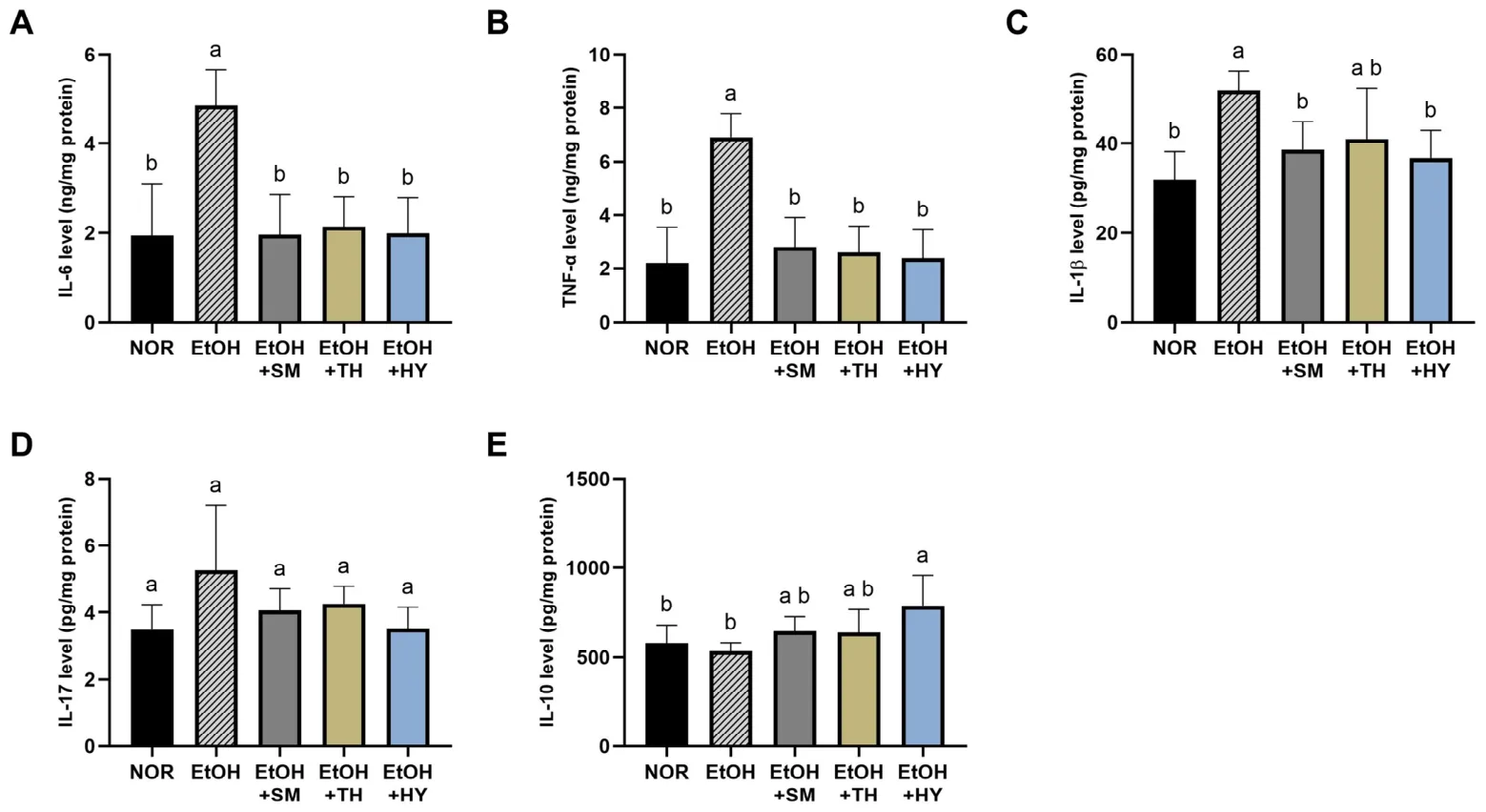
Effects of HY7804 on hepatic inflammatory cytokine levels in chronic-binge ethanol-fed mice. Levels of pro-inflammatory cytokines (**A**) IL-6, (**B**) TNF-α, (**C**) IL-1β, (**D**) IL-17 and anti-inflammatory cytokine (**E**) IL-10. Data are shown as mean ± SD. Statistical comparisons were performed using one-way ANOVA followed by Tukey’s multiple comparisons test. Groups sharing at least one letter do not differ significantly, whereas groups with no letters in common differ significantly (*p* < 0.05). IL-6, Interleukin-6; TNF-α, Tumor necrosis factor-alpha; IL-1β, Interleukin-1 beta; IL-17, Interleukin-17; IL-10, Interleukin-10; NOR, normal group; EtOH, ethanol-fed group; EtOH + SM, silymarin-treated ethanol-fed group; EtOH + TH, *L. helveticus* type strain ATCC15009-treated ethanol-fed group; EtOH + HY, *L. helveticus* HY7804-treated ethanol-fed group.

Hepatic inflammatory cytokine responses were examined following HY7804 administration in chronic-binge ethanol-fed mice. Hepatic IL-6 and TNF-α levels were significantly higher in the EtOH group than in the NOR group (Figure 7A,B; *p* < 0.001). Treatment with SM, TH, or HY7804 was associated with significantly lower levels of both cytokines relative to the EtOH group (*p* < 0.001), with values comparable to those of the NOR group. Hepatic IL-1β levels showed a similar increase following ethanol feeding (Figure 7C; *p* < 0.001). SM and HY7804 administration significantly reduced IL-1β levels compared with the EtOH group (*p* < 0.05), whereas the decrease observed following TH treatment was not statistically significant.

Hepatic IL-17 levels were not significantly different among the groups; however, the EtOH group showed a tendency toward increased IL-17 levels, whereas all treatment groups exhibited lower levels compared with the EtOH group (Figure 7D). HY7804 administration significantly increased hepatic IL-10 levels relative to the EtOH group (Figure 7E; *p* < 0.01), while SM and TH treatments showed no significant effects. These findings demonstrate that HY7804 influenced ethanol-associated hepatic cytokine responses, characterized by reduced pro-inflammatory cytokine changes and increased IL-10 expression in chronic-binge ethanol-fed mice.

### 2.9. Effect of HY7804 on Hepatic Apoptosis-Related Gene Expression in Chronic-Binge Ethanol-Fed Mice

**Figure 8:**
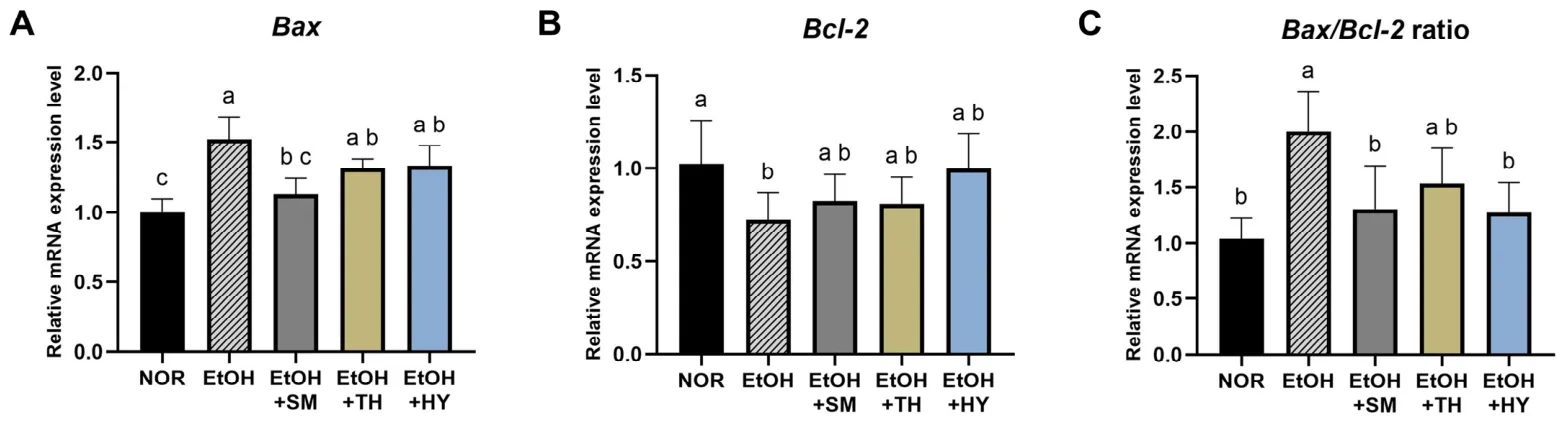
Hepatic expression of apoptosis-related genes following HY7804 administration in chronic-binge ethanol-fed mice. Relative mRNA levels of *Bax* (**A**), *Bcl-2* (**B**), and the *Bax*/*Bcl-2* ratio (**C**) are shown. Data are shown as mean ± SD. Statistical comparisons were performed using one-way ANOVA followed by Tukey’s multiple comparisons test. Groups sharing at least one letter do not differ significantly, whereas groups with no letters in common differ significantly (*p* < 0.05). *Bax*, Bcl-2 associated X; *Bcl-2*, B-cell lymphoma 2; NOR, normal group; EtOH, ethanol-fed group; EtOH + SM, silymarin-treated ethanol-fed group; EtOH + TH, *L. helveticus* type strain ATCC15009-treated ethanol-fed group; EtOH + HY, *L. helveticus* HY7804-treated ethanol-fed group.

To evaluate whether HY7804 modulates ethanol-induced apoptosis responses in the liver, the mRNA expression of apoptosis-related genes was analyzed in chronic-binge ethanol-fed mice. Hepatic *Bax* expression was significantly increased in the EtOH group compared with the NOR group (*p* < 0.001). Among the treatment groups, SM administration significantly reduced *Bax* expression relative to the EtOH group (*p* < 0.001), whereas TH and HY treatments showed a downward tendency without statistical significance. Conversely, ethanol exposure resulted in a significant reduction in hepatic *Bcl-2* expression compared with the NOR group (Figure 8B, *p* < 0.05). Although SM, TH, and HY administration increased *Bcl-2* expression compared with the EtOH group, these changes did not reach statistical significance. The *Bax*/*Bcl-2* ratio was markedly elevated following ethanol feeding (*p* < 0.001), while SM and HY treatments significantly decreased this ratio compared with the EtOH group (*p* < 0.01). TH showed an intermediate response without a significant difference from either the NOR or EtOH group. Collectively, these findings show that HY7804 reduced the ethanol-associated increase in the hepatic *Bax*/*Bcl-2* mRNA expression ratio. However, these transcriptional changes alone do not establish an effect of HY7804 on hepatocellular apoptosis.

## 3. Discussion

Alcohol-associated liver disease (ALD) is a progressive liver disorder driven by excessive alcohol exposure and is characterized by hepatic steatosis, inflammation and fibrosis, which may progress to cirrhosis [1]. The therapeutic limitations of ALD underscore the need for alternative strategies. Our results indicate that *Lactobacillus helveticus* HY7804 may offer protective activity against ethanol-induced hepatic injury, with its effects accompanied by changes in hepatic oxidative stress, inflammatory signaling, and apoptosis-related responses. Using ethanol-treated HepG2 cells and a chronic-binge ethanol-fed mouse model, we showed that HY7804 improved key features of alcohol-induced hepatic injury, including cytotoxicity, serum transaminase elevation, hepatic lipid accumulation, inflammatory cytokine production, and alterations in oxidative stress-related markers. Collectively, these observations suggest that HY7804 favorably influences multiple hepatic responses associated with ethanol-induced hepatic injury.

In the in vitro experiments, ethanol exposure markedly reduced HepG2 cell viability and increased LDH release, indicating ethanol-induced cytotoxicity. HY7804 treatment improved cell viability in a dose-dependent manner and reduced ethanol-induced LDH release, suggesting that HY7804 alleviated ethanol-induced cellular damage in HepG2 cells. These results indicate that HY7804 may contribute to the maintenance of hepatocellular integrity under ethanol-induced stress conditions.

Ethanol-induced hepatocellular injury is closely associated with inflammatory activation, and damaged hepatocytes can contribute to the hepatic inflammatory milieu by releasing pro-inflammatory mediators [25]. In this study, ethanol exposure increased the secretion of IL-6, IL-1β, IL-8 and TNF-α in HepG2 cells, indicating that ethanol-induced cellular stress was accompanied by an inflammatory cytokine response. HY7804 treatment reduced the secretion of these cytokines. Although HepG2 cells do not fully recapitulate the complex inflammatory environment of the liver, these findings indicate that HY7804 can attenuate ethanol-induced cytokine responses in hepatocyte-like cells.

In addition, ethanol exposure increased the expression of apoptosis-related genes, including *CASP9* and *CASP3*, in HepG2 cells. Caspase-9 is involved in the intrinsic apoptosis pathway, whereas caspase-3 acts as a key executioner caspase in the apoptotic process [26]. HY7804 treatment downregulated ethanol-induced *CASP9* and *CASP3* expression, suggesting that HY7804 may modulate apoptosis-related gene expression under ethanol-induced stress conditions. Taken together, these changes may be associated with the decreased cytotoxicity observed in the viability and LDH assays following HY7804 treatment.

To determine whether the cellular effects of HY7804 observed in vitro could be extended to an in vivo setting, HY7804 was further evaluated using a chronic-binge ethanol-fed mouse model. In this model, chronic-binge ethanol feeding induced hepatic enlargement, whereas HY7804 administration significantly reduced these changes. This suggests that HY7804 may mitigate ethanol-associated alterations in liver mass, which occurred in parallel with improvements in hepatic injury-related parameters. In contrast, spleen weight was not affected by ethanol feeding or HY7804 administration under the present experimental conditions.

Serum AST and ALT levels serve as established biochemical markers of hepatocellular injury [27]. Chronic-binge ethanol exposure significantly increased serum AST and ALT levels in our experimental model, supporting the occurrence of ethanol-associated hepatic injury. HY7804 significantly reduced both serum transaminase levels compared with the EtOH group, suggesting that HY7804 alleviated ethanol-associated hepatocellular damage. This finding is consistent with the reduced cytotoxicity observed in ethanol-treated HepG2 cells following HY7804 treatment.

Hepatic lipid accumulation is one of the characteristic early changes induced by excessive alcohol exposure [28]. In the present study, H&E staining indicated ethanol-associated steatotic changes in the liver, which appeared to be alleviated following HY7804 administration. Consistently, Oil Red O staining showed increased hepatic lipid deposition in the EtOH group, and quantitative analysis demonstrated that HY7804 significantly reduced Oil Red O-positive areas. These findings suggest that HY7804 reduced ethanol-associated hepatic lipid accumulation and improved related histological alterations in chronic-binge ethanol-fed mice.

Following the histological evidence of hepatic lipid accumulation, we next examined oxidative stress-related responses, given the central role of ethanol metabolism-driven ROS generation in ethanol-induced hepatic injury [4,5]. In the present study, chronic-binge ethanol feeding impaired hepatic antioxidant enzyme defense, as indicated by reduced catalase and SOD activities and decreased *Cat* and *Sod2* expression. HY7804 administration restored *Cat*, *Sod1* and *Sod2* expression and increased catalase and SOD activities, suggesting that HY7804 may reinforce enzymatic antioxidant defense under ethanol-induced oxidative stress conditions. In contrast, *Gpx1* expression was not significantly altered among the groups, suggesting that the antioxidant-related effects of HY7804 observed in this model may be more closely associated with catalase and SOD responses than with changes in *Gpx1* expression. As a major product generated during lipid peroxidation, MDA is widely used to assess oxidative damage in hepatic tissue [29]. Previous studies have reported that ethanol exposure increases hepatic MDA levels, indicating enhanced lipid peroxidation under ethanol-induced oxidative stress conditions [30,31]. In agreement with these reports, chronic-binge ethanol feeding elevated hepatic MDA levels in the present study, suggesting enhanced ethanol-associated lipid peroxidation. Conversely, HY7804 significantly reduced hepatic MDA levels, indicating attenuation of ethanol-induced oxidative lipid damage.

To further support the antioxidant-related effects of HY7804, we investigated the expression of Nrf2-related antioxidant genes. Nrf2 is an important transcriptional regulator involved in cellular defense against oxidative stress by controlling the expression of antioxidant and cytoprotective genes [32]. Among its downstream targets, *Hmox1* and *Gclc* contribute to heme degradation and glutathione biosynthesis, respectively [33,34]. In this study, chronic-binge ethanol feeding significantly downregulated hepatic *Nfe2l2* and *Gclc* expression, while *Hmox1* expression also showed a decreasing tendency. HY7804 administration increased the expression of *Hmox1*, *Nfe2l2*, and *Gclc*, suggesting that HY7804 may contribute to the recovery of ethanol-impaired Nrf2-related antioxidant gene expression. Together with the reduced MDA levels and restored catalase- and SOD-related antioxidant responses, these findings indicate that HY7804 treatment was associated with improved hepatic antioxidant responses under ethanol-induced oxidative stress conditions. However, because this study assessed mRNA expression rather than Nrf2 protein expression, nuclear translocation, or HO-1 protein levels, further studies incorporating protein-level validation and assessment of Nrf2 nuclear translocation are required to determine whether HY7804 directly influences Nrf2/HO-1 signaling activity.

Ethanol-induced oxidative stress may contribute to hepatic inflammation by activating redox-sensitive signaling pathways and promoting the production of pro-inflammatory cytokines [9,35]. In our chronic-binge ethanol-feeding model, hepatic IL-6, TNF-α, and IL-1β levels were elevated by ethanol exposure, whereas HY7804 administration significantly lowered their levels. Although the difference in IL-17 levels among the groups was not statistically significant, HY7804 treatment showed a downward trend relative to the EtOH group. In contrast, hepatic IL-10 levels were significantly increased following HY7804 administration. As an anti-inflammatory cytokine, IL-10 plays an important role in limiting excessive inflammatory responses. Collectively, the cytokine profile observed following HY7804 treatment was characterized by lower levels of pro-inflammatory mediators and increased IL-10, suggesting a shift toward a less inflammatory hepatic environment under chronic-binge ethanol exposure.

Sustained oxidative stress and inflammatory activation are closely linked to hepatocellular apoptosis, thereby contributing to ethanol-induced liver injury [36]. *Bax* is a pro-apoptotic factor, whereas *Bcl-2* counteracts apoptosis; therefore, the *Bax*/*Bcl-2* ratio reflects the relative shift toward a pro-apoptotic expression profile [37]. In our chronic-binge ethanol-fed mouse model, ethanol feeding significantly elevated the hepatic *Bax*/*Bcl-2* ratio, whereas HY7804 administration significantly reduced this increase without significantly altering individual *Bax* or *Bcl-2* expression levels. These results suggest that HY7804 may influence ethanol-associated apoptosis-related gene expression imbalance; however, further protein-level and histological analyses are required to determine whether HY7804 directly affects apoptotic processes.

Overall, HY7804 treatment was associated with reduced ethanol-induced hepatic injury, together with changes in inflammatory responses, antioxidant defense, lipid peroxidation, and apoptosis-related gene expression in the liver. The present study supports the potential of HY7804 as a functional probiotic ingredient for alleviating ethanol-associated hepatic damage. However, this study did not evaluate protein-level changes in Nrf2-related or apoptosis-related pathways, nor did it assess intestinal barrier function, gut microbiota composition, or endotoxin-related responses. Therefore, future studies are needed to determine whether HY7804-mediated protection involves gut-liver axis modulation and to identify HY7804-derived bioactive metabolites or strain-specific functional components responsible for its protective effects. Such investigations would help clarify the broader mechanisms underlying the hepatoprotective potential of HY7804 in ethanol-induced hepatic injury.

## 4. Materials and Methods

### 4.1. Lactobacillus helveticus Culture and Preparation

*Lactobacillus helveticus* HY7804 (HY7804), isolated from raw fresh milk obtained from domestic animals, was provided by R&BD Center of hy Co., Ltd. (Yongin, Republic of Korea) HY7804 was stored at −80 °C in MRS broth (BD Difco, Sparks, MD, USA) supplemented with 25% (*v*/*v*) glycerol (Sigma-Aldrich, St. Louis, MO, USA). *Lactobacillus helveticus* type strain ATCC15009 (American Type Culture Collection (ATCC), Manassas, VA, USA) served as the reference strain for the animal experiments. For preparation, both strains were grown anaerobically in MRS broth at 37 °C for 18 h and harvested by centrifugation at 3000 × *g* for 15 min. The resulting pellets were washed twice and resuspended in sterile PBS for the in vitro experiments. The lyophilized preparations used for the animal experiment were produced at the Probiotics Plant of hy Co., Ltd. (Pyeongtaek, Republic of Korea). The viable counts of the lyophilized preparations used for the animal study were determined by serial dilution and plate counting and were expressed as CFU/g. The viable count of the HY7804 preparation after lyophilization was 9.9 × 10^10^ CFU/g, while that of ATCC15009 was 5.8 × 10^10^ CFU/g. The preparations were stored at −20 °C until use. Periodic plate counts confirmed that the viable counts remained stable during storage and throughout the administration period. The amount administered was calculated based on the confirmed viable count to provide 1 × 10^9^ CFU/kg/day.

### 4.2. Cell Culture and Sample Treatment

HepG2 cells, a human hepatocellular carcinoma cell line, were obtained from the Korean Cell Line Bank (Seoul, Republic of Korea). Cells were maintained in Minimum Essential Medium (MEM; WELGENE, Gyeongsan, Republic of Korea) supplemented with 10% fetal bovine serum (Gibco, Waltham, MA, USA) and 1% penicillin/streptomycin (Gibco, Waltham, MA, USA) at 37 °C under a humidified atmosphere containing 5% CO_2_. For the experiments, cells were seeded in 6-well plates at 5 × 10^4^ cells/well and allowed to stabilize for 24 h. The medium was then replaced with fresh MEM without serum and antibiotics. For the cell viability assay, two separate experiments were conducted. First, HepG2 cells were treated with HY7804 at 10^5^–10^7^ CFU/mL for 4 h to evaluate the effects of HY7804 alone on cell viability. In a separate experiment, cells were pretreated with HY7804 at 10^5^–10^7^ CFU/mL for 4 h and subsequently exposed to 750 mM ethanol (Cat. No. 459844, Sigma-Aldrich, St. Louis, MO, USA) for 48 h to evaluate the protective effects of HY7804 against ethanol-induced cytotoxicity. The concentration of 750 mM ethanol was selected based on preliminary concentration-response experiments (Figure S1) and previous HepG2-based ethanol injury studies [38,39]. For the analyses of inflammatory cytokine production and apoptosis-related gene expression, cells were treated with HY7804 before ethanol exposure under the same conditions. A HY7804-only group was not included in the inflammatory cytokine or apoptosis-related gene expression analyses.

### 4.3. MTT Assay

HepG2 cells were plated in 96-well plates at 1 × 10^4^ cells/well. HY7804 suspended in PBS was applied at 10^5^, 10^6^, or 10^7^ CFU/mL. The bacterial cells were maintained in the culture during the 4-h pretreatment and subsequent ethanol exposure; no bacterial removal or medium replacement was performed between the HY7804 pretreatment and ethanol treatment. After the 4-h pretreatment, ethanol was added to obtain a final concentration of 750 mM, and the cells were maintained for a further 48 h. MTT solution was then added to each well at a final concentration of 0.5 mg/mL and incubated for 4 h. The MTT-containing medium was completely aspirated to remove non-adherent bacterial cells, followed by two washes with PBS. The formazan crystals retained within the adherent HepG2 cells were subsequently solubilized in 100 μL of dimethyl sulfoxide (DMSO; Sigma-Aldrich, St. Louis, MO, USA), and absorbance was recorded at 570 nm using a BioTek^®^ Synergy HT Microplate reader (Santa Clara, CA, USA).

### 4.4. LDH Assay

LDH activity released into the culture medium was quantified with the CytoTox 96 Non-Radioactive Cytotoxicity Assay Kit (Promega, Madison, WI, USA) following the supplier’s protocol. Absorbance was recorded at 490 nm.

### 4.5. Animal Experimental Design

Female C57BL/6 mice, 8 weeks of age and weighing 19–21 g, were purchased from Dooyeol Biotech (Seoul, Republic of Korea) and maintained at 21–23 °C and 45–65% relative humidity. After an acclimation period, the mice were randomly assigned to five groups (*n* = 6): a normal group (NOR, Lieber-DeCarli Regular Control diet, DYET #710027 (Dyets, Inc., Bethlehem, PA, USA)), an ethanol-fed group (EtOH, Lieber-DeCarli Ethanol Diet, DYET #710260((Dyets, Inc., Bethlehem, PA, USA))), ethanol-fed group treated with silymarin (EtOH + SM, positive control, 100 mg/kg/day) [13,40,41], ethanol-fed group treated with *L. helveticus* type strain ATCC15009 (EtOH + TH, 10^9^ CFU/kg/day) and ethanol-fed group treated with *L. helveticus* HY7804 (EtOH + HY, 10^9^ CFU/kg/day). The NOR group was pair-fed an isocaloric control diet matched to the dietary intake of the ethanol-fed groups.

Silymarin and probiotics were orally administered for 10 days prior to and throughout the ethanol feeding period. The NOR and EtOH groups were orally administered an equivalent volume of saline. The chronic-binge ethanol feeding regimen was established based on the National Institute on Alcohol Abuse and Alcoholism (NIAAA) model and previously reported ethanol-feeding protocols [10,11,13]. Ethanol was provided using a Lieber-DeCarli ethanol liquid diet. To facilitate adaptation to ethanol exposure and minimize potential reductions in food intake associated with abrupt exposure to a high ethanol concentration, the ethanol concentration was gradually increased during the initial 5 days (1% for 2 days, 2% for 2 days, and 4% for 1 day). The mice were then maintained on a 5% ethanol-containing diet for 10 days. On day 16, mice were additionally gavaged a single oral dose of ethanol (5 g/kg). The NOR group was gavaged an isocaloric maltodextrin solution instead of ethanol. Nine hours after gavage, the mice were sacrificed and blood and liver tissue were collected. Serum was prepared by centrifugation at 3000× *g* for 20 min. All animal procedures were approved by the Institutional Animal Care and Use Committee of hy Co., Ltd., Seoul, Republic of Korea (AEC-2026-0006-Y; approved 21 April 2026).

### 4.6. Serum Biochemical Analysis

Serum alanine aminotransferase (ALT) and aspartate aminotransferase (AST) levels were determined by Dooyeol Biotech (Seoul, Republic of Korea).

### 4.7. Histological Examination

For histological analysis, liver tissues were fixed in 10% formalin (Sigma-Aldrich, St. Louis, MO, USA), and paraffin-embedded sections were prepared and stained with hematoxylin and eosin (H&E) by Dooyeol Biotech (Seoul, Republic of Korea). For Oil Red O staining, separate tissue samples were embedded in optimal cutting temperature (OCT) compound to generate frozen blocks. Sections with a thickness of 8–10 μm were obtained using a cryostat and subjected to Oil Red O staining. Five randomly selected fields per sample were imaged at 100× magnification. The Oil Red O-positive area was determined using ImageJ software (version 1.54k) and calculated as the percentage of stained area relative to the total tissue area.

### 4.8. Measurement of Antioxidant Enzymes and Lipid Peroxidation

For the analysis of antioxidant enzyme activities, liver tissues were prepared in accordance with the respective manufacturers’ protocols. Briefly, the tissues were homogenized in the assay buffer and centrifuged, and the resulting supernatants were collected for measurement of catalase and superoxide dismutase (SOD) activities using the Catalase Assay Kit and SOD Assay Kit (BO-CAT-400 and BO-SOD-250, respectively; BIOMAX, Guri-si, Gyeonggi-do, Republic of Korea). Catalase and SOD activities were normalized to the total protein concentration of the corresponding liver homogenates.

For MDA analysis, liver tissues were washed with PBS, homogenized in the lysis buffer provided with the Lipid Peroxidation (MDA) Assay Kit (ab118970, Abcam, Cambridge, UK), and centrifuged according to the manufacturer’s instructions. The resulting supernatants were collected for hepatic MDA quantification. MDA levels were normalized to tissue weight and expressed as nmol/mg tissue.

### 4.9. Measurement of Cytokine Levels

The levels of IL-6, IL-1β, TNF-α and IL-8 in the culture supernatants were quantified using the BD OptEIA™ Human ELISA Set (IL-6, IL-1β, TNF-α, and IL-8; BD555220, BD557953, BD555212 and BD555244; BD Biosciences, San Diego, CA, USA) according to the manufacturer’s instructions.

For hepatic cytokine analysis, liver tissues were homogenized in ice-cold PBS containing a protease inhibitor cocktail. The homogenates were centrifuged at 10,000× *g* for 15 min at 4 °C, and the resulting supernatants were collected for cytokine measurements. Hepatic IL-6, TNF-α, and IL-10 levels were determined using the BD OptEIA™ Mouse ELISA Sets (IL-6, TNF-α, and IL-10; BD555240, BD555268, and BD555252, respectively; BD Biosciences, San Diego, CA, USA). IL-17 and IL-1β concentrations were measured using the Mouse IL-17 Quantikine ELISA Kit (M1700, R&D Systems, Minneapolis, MN, USA) and Mouse IL-1β Uncoated ELISA Kit (88-7013A, Invitrogen, Waltham, MA, USA), respectively, following the manufacturers’ instructions. Total protein concentrations were determined using the bicinchoninic acid (BCA) assay, and cytokine measurements were normalized to the corresponding total protein content. Data were expressed as pg/mg protein or ng/mg protein.

### 4.10. Quantitative Real-Time PCR (qPCR) Analysis

Total RNA was extracted from HepG2 cells and mouse liver tissues with the easy-spin Total RNA Extraction Kit (iNtRON Biotechnology, Seoul, Republic of Korea) following the manufacturer’s instructions. The concentration and purity of extracted RNA were determined using a microvolume spectrophotometer. Equal amounts of total RNA were used for cDNA synthesis using the Omniscript Reverse Transcription Kit (Qiagen, Hilden, Germany) according to the manufacturer’s instructions. qPCR analysis was carried out on a QuantStudio 6-Flex Real-time PCR System (Applied Biosystems, Foster City, CA, USA) using TaqMan Gene Expression Master Mix and TaqMan Gene Expression Assays (Applied Biosystems). Each qPCR reaction was performed in a final volume of 20 μL containing 10 μL of TaqMan Gene Expression Master Mix, 1 μL of the corresponding TaqMan Gene Expression Assay, 2 μL of cDNA, and 7 μL of nuclease-free water. The thermal cycling conditions consisted of an initial denaturation at 95 °C for 10 min, followed by 40 cycles of denaturation at 95 °C for 15 s and annealing/extension at 60 °C for 60 s. Table 1 summarizes the target genes together with their corresponding TaqMan Gene Expression Assay IDs. For normalization, *GAPDH* was used as the reference gene in human HepG2 cells, whereas *Gapdh* served as the reference gene in mouse liver tissues. Relative transcript levels were determined using the comparative cycle threshold method (2^−ΔΔCt^).

### 4.11. Statistical Analysis

Statistical comparisons among multiple groups were conducted using one-way analysis of variance (ANOVA) followed by Tukey’s post hoc test. Analyses were performed with GraphPad Prism 10. Results are expressed as mean ± standard deviation (SD), and *p* < 0.05 was considered indicative of statistical significance.

## 5. Conclusions

Overall, the findings support a protective effect of *L. helveticus* HY7804 against ethanol-associated cellular injury in HepG2 cells and hepatic injury in chronic-binge ethanol-fed mice. HY7804 improved ethanol-associated cellular damage, serum transaminase elevation, hepatic lipid deposition, and histopathological abnormalities. These protective effects were accompanied by reduced hepatic inflammatory responses, improved antioxidant-related responses, decreased lipid peroxidation, and modulation of apoptosis-related gene expression. Taken together, this study highlights HY7804 as a potential probiotic material for alcohol-related liver health, while further studies are warranted to define its active components and clarify its translational relevance.

## Figures and Tables

**Table 1:** TaqMan probes used in vitro and animal experiments.

Gene Symbol	Gene Name	Assay ID
*GAPDH*	Glyceraldehyde-3-phosphate dehydrogenase	Hs99999905_m1
*CASP3*	Caspase-3	Hs00234387_m1
*CASP9*	Caspase-9	Hs00962278_m1
*Gapdh*	Glyceraldehyde-3-phosphate dehydrogenase	Mm99999915_g1
*Cat*	Catalase	Mm00437992_m1
*Sod1*	Superoxide dismutase 1, soluble	Mm01344233_g1
*Sod2*	Superoxide dismutase 2, soluble	Mm01313000_m1
*Gpx1*	Glutathione peroxidase 1	Mm00656767_g1
*Hmox1*	Heme oxygenase 1	Mm00516005_m1
*Nfe2l2*	Nuclear factor, erythroid derived 2, like 2	Mm00477784_m1
*Gclc*	Glutamate-cysteine ligase, catalytic subunit	Mm00802658_m1
*Bax*	BCL2-associated X protein	Mm00432051_m1
*Bcl-2*	BCL2, apoptosis regulator	Mm00477631_m1

## Data Availability

The data presented in this study are available from the corresponding author upon reasonable request.

## Supplementary Materials

The following supporting information can be downloaded at: https://www.mdpi.com/article/10.3390/ijms27188058/s1.

## References

[1] Åberg F., Jiang Z.G., Cortez-Pinto H., Männistö V. (2024). Alcohol-associated liver disease—Global epidemiology. Hepatology.

[2] Sosnowski K., Kucharski R., Przybyłkowski A. (2026). Efficacy of probiotic treatment in alcoholic liver disease: A systematic review of animal studies. Nutrients.

[3] Jophlin L.L., Singal A.K., Bataller R., Wong R.J., Sauer B.G., Terrault N.A., Shah V.H. (2023). ACG clinical guideline: Alcohol-associated liver disease. Am. J. Gastroenterol..

[4] Lai W., Zhang J., Sun J., Min T., Bai Y., He J., Cao H., Che Q., Guo J., Su Z. (2024). Oxidative stress in alcoholic liver disease, focusing on proteins, nucleic acids, and lipids: A review. Int. J. Biol. Macromol..

[5] Lee J.-Y., Jee Y.-M., Yang K., Ryu T. (2025). Alcohol-induced oxidative stress and gut–liver–brain crosstalk: Expanding the paradigm from ALD to MetALD. Antioxidants.

[6] Louvet A., Mathurin P. (2015). Alcoholic liver disease: Mechanisms of injury and targeted treatment. Nat. Rev. Gastroenterol. Hepatol..

[7] Diesen D.L., Kuo P.C. (2010). Nitric oxide and redox regulation in the liver: Part I. General considerations and redox biology in hepatitis. J. Surg. Res..

[8] Sun J., Hong Z., Shao S., Li L., Yang B., Hou Y., Wang H., Xu Y., Zhang Q., Pi J. (2021). Liver-specific Nrf2 deficiency accelerates ethanol-induced lethality and hepatic injury in vivo. Toxicol. Appl. Pharmacol..

[9] Wang H.J., Gao B., Zakhari S., Nagy L.E. (2012). Inflammation in alcoholic liver disease. Annu. Rev. Nutr..

[10] Bertola A., Mathews S., Ki S.H., Wang H., Gao B. (2013). Mouse model of chronic and binge ethanol feeding (the NIAAA model). Nat. Protoc..

[11] Appolonia C.N., Wolf K.M., Zawatsky C.N., Cinar R. (2022). Chronic and binge alcohol ingestion increases truncated oxidized phosphatidylcholines in mice lungs due to increased oxidative stress. Front. Physiol..

[12] Gao B., Xu M.-J., Bertola A., Wang H., Zhou Z., Liangpunsakul S. (2017). Animal models of alcoholic liver disease: Pathogenesis and clinical relevance. Gene Expr..

[13] Kim W.-G., Kim H.I., Kwon E.K., Han M.J., Kim D.-H. (2018). Lactobacillus plantarum LC27 and Bifidobacterium longum LC67 mitigate alcoholic steatosis in mice by inhibiting LPS-mediated NF-κB activation through restoration of the disturbed gut microbiota. Food Funct..

[14] Fulham M.A., Mandrekar P. (2016). Sexual dimorphism in alcohol induced adipose inflammation relates to liver injury. PLoS ONE.

[15] Sanders M.E., Gibson G., Gill H.S., Guarner F. (2007). Probiotics: Their Potential to Impact Human Health.

[16] Gatti M., Trivisano C., Fabrizi E., Neviani E., Gardini F. (2004). Biodiversity among Lactobacillus helveticus strains isolated from different natural whey starter cultures as revealed by classification trees. Appl. Environ. Microbiol..

[17] Bertani G., Bassi D., Gatti M., Cocconcelli P.S., Neviani E. (2018). Draft genome sequence of Lactobacillus helveticus strain Lh 12 isolated from natural whey starter. Genome Announc..

[18] Chelladhurai K., Ayyash M., Turner M.S., Kamal-Eldin A. (2023). Lactobacillus helveticus: Health effects, current applications, and future trends in dairy fermentation. Trends Food Sci. Technol..

[19] Hsieh P.-S., Chen C.-W., Kuo Y.-W., Ho H.-H. (2021). Lactobacillus spp. reduces ethanol-induced liver oxidative stress and inflammation in a mouse model of alcoholic steatohepatitis. Exp. Ther. Med..

[20] Lv J., Lang G., Wang Q., Zhao W., Shi D., Zhou Z., Shen Y., Xia H., Han S., Li L. (2024). Lactobacillus helveticus attenuates alcoholic liver injury via regulation of gut microecology in mice. Microb. Biotechnol..

[21] Tian F., Chi F., Wang G., Liu X., Zhang Q., Chen Y., Zhang H., Chen W. (2015). Lactobacillus rhamnosus CCFM1107 treatment ameliorates alcohol-induced liver injury in a mouse model of chronic alcohol feeding. J. Microbiol..

[22] Kim H., Lee K., Kim J.-Y., Shim J.-J., Lim J., Kim J.-Y., Lee J.-L. (2023). Lactobacillus helveticus isolated from raw milk improves liver function, hepatic steatosis, and lipid metabolism in non-alcoholic fatty liver disease mouse model. Microorganisms.

[23] Kim H., Jeon H.-J., Jeong J.-W., Lee K., Gwon H., Lee D., Kim J.-Y., Shim J.-J., Lee J.-H. (2025). Lactobacillus helveticus HY7804 Modulates the Gut–Liver Axis to Improve Metabolic Dysfunction-Associated Steatotic Liver Disease in a Mouse Model. Int. J. Mol. Sci..

[24] Malik A., Malik M., Qureshi S. (2024). Effects of silymarin use on liver enzymes and metabolic factors in metabolic dysfunction-associated steatotic liver disease: A systematic review and meta-analysis. Can. Liver J..

[25] Yang Y.M., Cho Y.E., Hwang S. (2022). Crosstalk between oxidative stress and inflammatory liver injury in the pathogenesis of alcoholic liver disease. Int. J. Mol. Sci..

[26] Dho S.H., Cho M., Woo W., Jeong S., Kim L.K. (2025). Caspases as master regulators of programmed cell death: Apoptosis, pyroptosis and beyond. Exp. Mol. Med..

[27] Kwo P.Y., Masuoka H.C., Schaefer E.A., Friedman L.S. (2026). Evaluation of abnormal liver biochemical test results. Gastroenterology.

[28] Jeon S., Carr R. (2020). Alcohol effects on hepatic lipid metabolism. J. Lipid Res..

[29] Lugo R., Gutiérrez-Solis A.L., Jimeno-Figueroa R.E., Góngora-Chan P., Vera-Aviles M., Williams-Jacquez D., Chaurand-Lara M., Valdivieso-Jimenez J.A., Medina-Vera I., Guevara-Cruz M. (2026). Malondialdehyde as a Predictor of Disease Severity and Cardiovascular Risk in Population with Metabolic Dysfunction-Associated Steatotic Liver Disease. Metabolites.

[30] Sampey B.P., Korourian S., Ronis M.J., Badger T.M., Petersen D.R. (2003). Immunohistochemical characterization of hepatic malondialdehyde and 4-hydroxynonenal modified proteins during early stages of ethanol-induced liver injury. Alcohol. Clin. Exp. Res..

[31] Roig R., Cascón E., Arola L., Bladé C., Salvadó M.J. (2000). Effects of chronic wine and alcohol intake on glutathione and malondialdehyde levels in rats. Nutr. Res..

[32] He F., Ru X., Wen T. (2020). NRF2, a transcription factor for stress response and beyond. Int. J. Mol. Sci..

[33] Yeudall S., Upchurch C.M., Leitinger N. (2024). The clinical relevance of heme detoxification by the macrophage heme oxygenase system. Front. Immunol..

[34] Mishra M., Zhong Q., Kowluru R.A. (2014). Epigenetic modifications of Nrf2-mediated glutamate–cysteine ligase: Implications for the development of diabetic retinopathy and the metabolic memory phenomenon associated with its continued progression. Free. Radic. Biol. Med..

[35] Ambade A., Mandrekar P. (2012). Oxidative stress and inflammation: Essential partners in alcoholic liver disease. Int. J. Hepatol..

[36] Hoek J.B., Pastorino J.G. (2002). Ethanol, oxidative stress, and cytokine-induced liver cell injury. Alcohol.

[37] Putri G.F.T. (2025). Bax/Bcl-2 ratio as the golden marker of apoptosis: Molecular mechanisms and regulatory pathways. Int. J. Cell Biomed. Sci..

[38] Chen M.-F., Gong F., Zhang Y.Y., Li C., Zhou C., Hong P., Sun S., Qian Z.-J. (2019). Preventive effect of YGDEY from tilapia fish skin gelatin hydrolysates against alcohol-induced damage in HepG2 cells through ROS-mediated signaling pathways. Nutrients.

[39] Qian Z.J., Chen M.F., Chen J., Zhang Y., Zhou C., Hong P., Yang P. (2021). Intracellular ethanol-mediated oxidation and apoptosis in HepG2/CYP2E1 cells impaired by two active peptides from seahorse (*Hippocampus kuda* bleeler) protein hydrolysates via the Nrf2/HO-1 and akt pathways. Food Sci. Nutr..

[40] Papackova Z., Heczkova M., Dankova H., Sticova E., Lodererova A., Bartonova L., Poruba M., Cahova M. (2018). Silymarin prevents acetaminophen-induced hepatotoxicity in mice. PLoS ONE.

[41] Kim S.-M., Diao W.-J., An W., Kim H.-J., Lim H.-J., Kim K.-N., Bae G.-W., Kang J.-S. (2022). Effect of porcine placental extract mixture on alcohol-induced hepatotoxicity in rats. Curr. Issues Mol. Biol..

