# Supplementary material for: Lactobacillus helveticus HY7804 Alleviates Ethanol-Induced Liver Injury and Modulates Oxidative Stress and Inflammatory Responses in a Chronic-Binge Ethanol-Fed Mouse Model
Source: Int J Mol Sci. 2026 Sep 10;27(18):8058. doi: 10.3390/ijms27188058 (PMC13606802; doi:10.3390/ijms27188058)
Supplement: Supplementary file 1 [file ijms-27-08058-s001.zip › ijms-4539751-supplementary.pdf]

Supplementary file

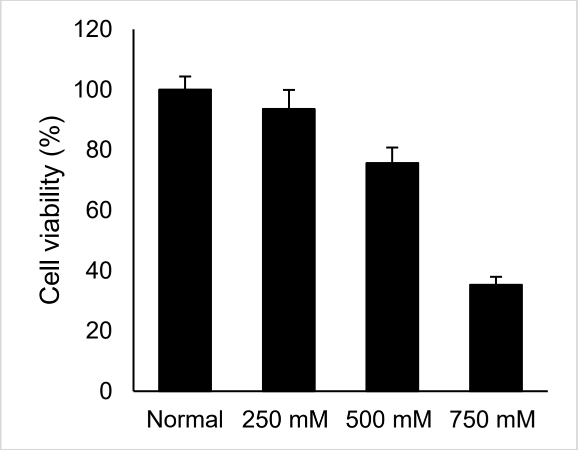

Figure S1. Preliminary evaluation of ethanol concentration for HepG2 cell injury induction. HepG2 cells were treated with different concentrations of ethanol (250, 500, and 750 mM) for 48 h, and cell viability was determined by the MTT assay. Data are expressed as mean ± SD.

Table S1. Evaluation of potential interference of HY7804 with the MTT assay.

Condition	Description	Absorbance at 570 nm
Blank	Medium + MTT	0.049 ± 0.001
HY7804 alone	HY7804 + medium + MTT (without HepG2 cells)	0.050 ± 0.001
HepG2 Control	HepG2 cells + MTT	0.500 ± 0.015
HY7804-treated HepG2	HepG2 cells + HY7804 + MTT	0.537 ± 0.020

HY7804 (10^7 CFU/mL) was incubated with MTT reagent in the absence of HepG2 cells to determine whether bacterial metabolic activity contributed to formazan formation. Absorbance values were measured under the same experimental conditions used for the HepG2 cell viability assay. Data are expressed as mean ± SD.

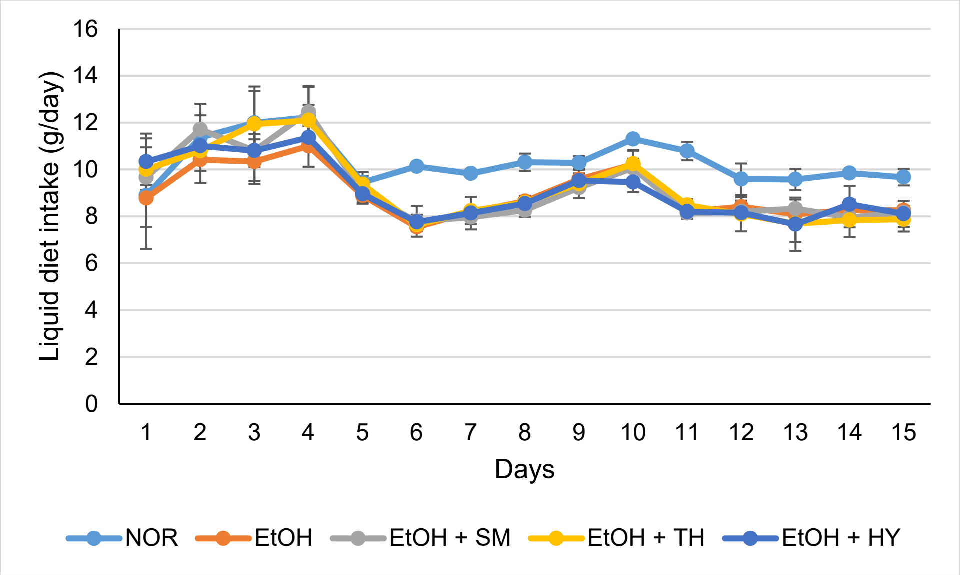

Figure S2. Daily liquid diet intake during the ethanol-feeding period. Daily intake of the Lieber-DeCarli liquid diet was monitored throughout the stepwise ethanol-feeding and subsequent 5% ethanol-feeding periods. Data are expressed as mean ± SD.
